# Familial Aggregation and Segregation Analysis in Families Presenting Autoimmunity, Polyautoimmunity, and Multiple Autoimmune Syndrome

**DOI:** 10.1155/2015/572353

**Published:** 2015-11-30

**Authors:** John Castiblanco, Juan Camilo Sarmiento-Monroy, Ruben Dario Mantilla, Adriana Rojas-Villarraga, Juan-Manuel Anaya

**Affiliations:** ^1^Center for Autoimmune Diseases Research (CREA), School of Medicine and Health Sciences, Universidad del Rosario, Carrera 24 No. 63-C-69, Bogotá, Colombia; ^2^Doctoral Program in Biomedical Sciences, Universidad del Rosario, Bogotá, Colombia

## Abstract

Studies documenting increased risk of developing autoimmune diseases (ADs) have shown that these conditions share several immunogenetic mechanisms (i.e., the autoimmune tautology). This report explored familial aggregation and segregation of AD, polyautoimmunity, and multiple autoimmune syndrome (MAS) in 210 families. Familial aggregation was examined for first-degree relatives. Segregation analysis was implemented as in S.A.G.E. release 6.3. Data showed differences between late- and early-onset families regarding their age, age of onset, and sex. Familial aggregation of AD in late- and early-onset families was observed. For polyautoimmunity as a trait, only aggregation was observed between sibling pairs in late-onset families. No aggregation was observed for MAS. Segregation analyses for AD suggested major gene(s) with no clear discernible classical known Mendelian transmission in late-onset families, while for polyautoimmunity and MAS no model was implied. Data suggest that polyautoimmunity and MAS are not independent traits and that gender, age, and age of onset are interrelated factors influencing autoimmunity.

## 1. Introduction

Autoimmune diseases (ADs) are responsible for a substantial amount of disability and morbidity worldwide. Although their epidemiology varies according to individual conditions, collectively, autoimmune prevalence is at least 5% in the general population and is one of the major causes of premature mortality in young and middle aged women [1].

As heterogeneous diseases, ADs develop from the cumulative effect of diverse events on the immune system [2]. It is clear that ADs do not begin at the time of clinical appearance but rather many years before. A common origin for diverse ADs is sustained by three levels of evidence [3]: the first comes from clinical observations indicating the possible shift from one disease to another or to the fact that more than one AD may coexist in a single patient (i.e., polyautoimmunity) [4–8] or in the same family (i.e., familial autoimmunity) [9]; a second level of evidence refers to known shared pathophysiological mechanisms between ADs [10, 11]. Epidemiological studies show correlations among certain ADs, linking epidemiological observations to physiopathological evidence for AD might contribute to our knowledge for the shared etiological and immunogenetic mechanisms [2]; and a third level of evidence corresponds to the evidence implying common genetic factors [7]. The importance of this concept focuses on the probability of having multiple ADs simultaneously in one patient, which goes beyond epidemiologic inferences.

Numerous genetic factors are established to be important contributors to susceptibility in developing ADs based on several findings including the examination of the concordance rates between relatives for many autoimmune diseases (ADs) [12]. However, due to their multifactorial and polygenic nature, accompanied by differential penetrance influenced by environmental factors and genetic heterogeneity among populations [13, 14], untangling of the genetic determinants defining their outcome and onset has proven to be extremely challenging. Likewise, data showing the existence of different ADs within a single family or within the same individual suggest a combination of genetic defects that may predispose individuals to different ADs sharing common pathogenic pathways [15].

Therefore, family history of ADs should be considered when performing genetic analysis as this new approach incorporates all accepted pathologies for which evidence suggests an autoimmune origin. Families with multiple affected relatives appear to share common risk alleles with sporadic patients but may have a higher genetic load. A consequence of the polygenic model for complex diseases is that patients are inevitably highly heterogeneous in terms of the particular set of risk alleles they carry. It has been suggested that this may translate in different genetically determined disease mechanisms in subgroups of patients or a common disease mechanism that is complemented by additional pathways that are more or less predominant in different subgroups [16]. Familial approaches have documented the clustering of certain ADs among the relatives of individuals who have rheumatoid arthritis (RA), multiple sclerosis (MS), systemic lupus erythematosus (SLE), and type 1 diabetes mellitus (T1D) among other diseases [17–23].

ADs are not inherited in a simple, classical Mendelian way but have instead a complex or a yet uncharacterized mode of inheritance [13, 24]. Bias et al. were the first to consider a single major gene conferring susceptibility for autoimmunity and suggested an autosomal dominant inheritance pattern with penetrance of approximately 92% in females and 9% in males [13]. In addition, Arcos-Burgos et al. showed the presence of a dominant major gene and strong environmental effects as the most parsimonious model of segregation for VIT [25]. On the other hand, when analyzing RA together with other ADs, a mixed model fitted the data significantly better than the major gene or polygenic models [26].

The clinical evidence of the autoimmune tautology highlights the cooccurrence of distinct ADs within an individual [27]. ADs coexistence in a single individual has led researchers to consider different terms like autoimmune diathesis [28] or kaleidoscope of autoimmunity [29] both of which point to a common genetic background of ADs [6]. In an effort to understand and further support the commonality of autoimmunity as a trait among ADs, the present study examined the dynamics of familial aggregation and segregation in AD, polyautoimmunity, and multiple autoimmune syndrome (MAS) in well-defined and characterized patients and their relatives from Colombia, South America.

## 2. Materials and Methods

### 2.1. Study Population and Family Collection

**Figure 1:**
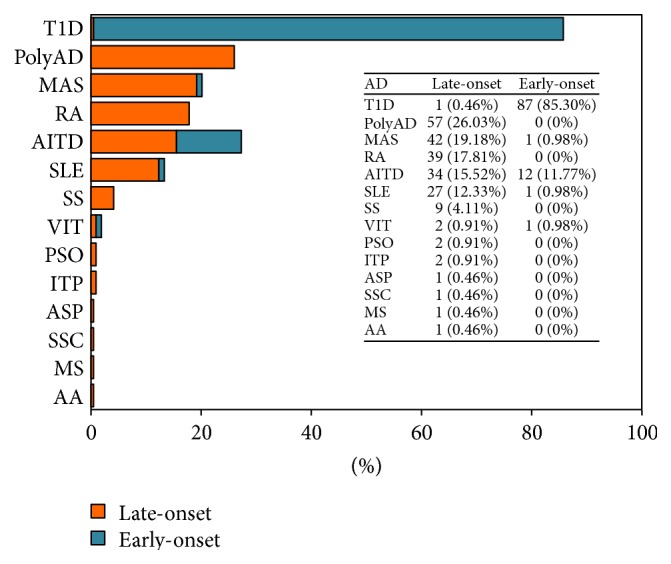
Frequency and distribution of autoimmune disease (AD) in late-onset and early-onset families included in this report. For analytical purposes, families were divided into two types: late-onset (i.e., families where a proband presents a late-onset AD) and early-onset (i.e., T1D families) (Figure 1).

This study sample consisted of multiplex families of varying size ascertained through patients treated at the Center for Autoimmune Diseases Research (CREA) in Medellin and Bogotá at the University of Rosario, Colombia (Table 1). (i) Each recruited family presented a proband with at least one AD according to validated international classification criteria; (ii) each recruited family presented at least one family member with polyautoimmunity (i.e., cooccurrence of distinct ADs within an individual); (iii) each recruited family presented evidence of familial autoimmunity (i.e., different ADs within members of a nuclear family); and (iv) each other affected individual presented a well-defined autoimmune phenotype (i.e., fulfillment of international classification criteria in first-degree relatives (FDRs)). Moreover, families in which the proband presented with T1D were included and used as early-onset AD families (Figure 1). FDRs were defined as parents and siblings.

Patients with AD, polyautoimmunity, and MAS fulfilled validated classification criteria and were part of a multicenter cohort followed at the CREA. Their information on demographics and cumulative clinical manifestations over the course of disease were obtained by both chart review and discussion with the patient and were collected in a standard data collection form. Only relatives of Colombian patients were included and interviewed, following the methodology described by Priori et al. [30], using a standardized questionnaire that incorporates demographics and medical information including a check-point list of 18 ADs [21]. In order to avoid ascertainment bias, the diagnosis of any AD was only considered reliable and consequently registered if made by a certified physician (i.e., internist, endocrinologist, or rheumatologist) and confirmed by chart review or verification during discussion with the relative. All patients fulfilled the diagnostic classification criteria proposed per disease as previously applied [6, 21].

In T1D families, recruited cases were children all of whom fulfilled the diagnostic classification criteria proposed by the American Diabetes Association (ADA) [31] and had been previously described [32] (Table 1). Their information on demographics and cumulative clinical manifestations over the course of disease were obtained by both chart review and discussion with the patient and were collected in a standard data collection form. A total of 87 patients with T1D were analyzed and their relatives were included (Table 1).

For individuals (i.e., probands and FDR) with thyroid disorders, anti-thyroglobulin and anti-thyroperoxidase antibodies were measured by enzyme-linked immunosorbent assay (QUANTA Lite, INOVA Diagnostics, San Diego, CA, USA). Only patients with positive antibody profile for autoimmune thyroid disease (AITD) were included for analysis. Exclusion criteria were preexisting hematological diseases and hepatitis B virus, hepatitis C virus, or human immunodeficiency virus infections. As for the family characteristics in our population, most of them are nuclear and at least 30% are multigenerational [33, 34]. The great majority of our country households still contain related persons. In addition, all family members participating in this study were living in the same city and approved informed consent in order to participate in the present study. This research is being carried out in accordance with Resolution number 008430 of 1993 issued by the Ministry of Health of the Republic of Colombia and was classified as a minimal risk research. The Ethics Committee of Universidad del Rosario approved the present project.

### 2.2. Statistical and Genetic Data Analysis

Data was managed and stored using the R software version 3.1.1 [35] and Excel spreadsheets. Results are presented as means ± standard deviation (SD) and minimum/maximum and/or in percentages. Comparison between means was performed by Student's *t*-test and those between percentages by the *χ* ^2^ test and two-sided Fisher's exact test, where appropriate. A *p* value of less than 0.05 was considered as statistically significant.

The present study included information on (i) sex, (ii) autoimmunity affection status defined as affected, unaffected, or unknown for AD (i.e., having at least one AD), polyautoimmunity (i.e., having at least two ADs), and MAS (i.e., having three or more ADs), and (iii) family/pedigree relationships. Estimation of the distributions of relationship types and affection status among relatives pairs were performed using the Statistical Analysis for Genetic Epidemiology (S.A.G.E.) program PEDINFO, release 6.3 [36]. Where necessary, dummy individuals were added to families for the purpose of connecting relatives within pedigrees, and the affection status for such dummy individuals was set to missing and thus they were not used in the analyses.

*Familial Aggregation Analysis*. Recurrent risk ratios (*λ*_*R*_) were calculated for first-degree relatedness (parent/offspring and sibling/sibling pairs) using the formula *λ*_*R*_ = *K*_Relative_/*K*, where *K*_Relative_ (*K*_*R*_) is the prevalence for a specific degree of relatedness in the sample and *K* is the mean prevalence in the population [37] and/or the previously reported *K* in specific pairs of relatives in the same population [21]. Information about the prevalence of ADs in our population is not clear and available; for this matter prevalence values in the range of 0.1%–0.5% were chosen as reported in the literature [1, 38–45]. Therefore, 0.5% (5/1000 individuals) for AD and 2.5% (25/1000 individuals) for all ADs taken together were selected as putative population prevalence as previously reported [1, 21, 38–45]. These methods were extended to ascertain whether or not clustering of two or more autoimmune disorders in relatives increased the probability or the risk for the presence of the disorder in the affected proband.

*Familial Segregation Analysis*. Analyses on 210 single ascertained pedigrees (Table 1) to identify the most plausible model explaining the segregation of AD, polyautoimmunity, and MAS in late-onset (non-T1D families) and early-onset families (T1D families) were performed for a binary trait as implemented in SEGREG S.A.G.E. release 6.3 (Table 2). SEGREG uses maximum-likelihood methods to estimate the parameters of mathematical models of disease occurrence in families. Each model assumes that the presence (or absence) of a putative disease allele influences susceptibility to the trait and applies the regressive multivariate logistic model allowing us to include available covariates into the fitted models.

The fitted models assumed that the likelihood for any two individuals presenting with the phenotype and having the major type over nuclear families is independent. Consequently, the susceptibility (marginal probability) that any pedigree member has a particular phenotype is the same for all members who have the same values of any covariates in the model. This susceptibility is given the cumulative logistic function *λ* = *e* ^*θy*^/(1 + *e* ^*θy*^), where *y* is the affection status phenotype of *i*th individual and *θ* is the logit of the susceptibility for *i*th individual defined as *θ*(*i*) = log⁡[*p*(*Y* = 1)/1 − *p*(*Y* = 1)] = *βg* + *φX*, where *β* is the baseline parameter, *g* is the susceptibility type and *X* is the covariate vector.

Analyses were performed by estimating the following parameters: type frequencies Ψ_*u*_ (*u* = AA, AB, BB): if the type frequencies were in Hardy-Weinberg equilibrium proportions, they were defined in terms of *q*_A_ (frequency of allele A); transmission probabilities *τ*_*u*_ (the probability that a parent of type *u* transmits allele A to an offspring: under Mendelian transmission, *τ*_AA_ = 1, *τ*_AB_ = 0.5, and *τ*_BB_ = 0); and baseline parameter *β*, which can be sex dependent and/or type dependent. Sporadic/environmental and genetic models that were considered in assessing type of familial association and possible evidence of transmission of major effect are shown in Table 2.

Every model was tested against the likelihood of the general (unrestricted) model, in which all parameters were unrestricted and allowed to fit the empirical data. The estimated model hypotheses of transmission were as follows: major gene type, Mendelian dominant, Mendelian recessive, Mendelian additive, random environmental effect, codominant, and no transmission (Table 2). A likelihood ratio test (LRT) was used to test the significance of the departure from a specified null hypothesis model using the asymptotic properties of the LRT distributed as chi-square distribution with degrees of freedom equal to the difference in the number of parameters estimated in both models. Using this test, a significant chi-square test indicates that the submodel tested can be rejected at the given alpha level, which means the hypothesized model does not fit the data. Models were also compared using Akaike's information criterion (AIC), which is defined as AIC = −2ln⁡*L* + 2*x* (number of parameters estimated). A lower value of AIC represents a better fitting model.

## 3. Results

In this study, 127 late-onset diseases and 83 early-onset families were examined. The general statistics of the pedigrees are disclosed in Table 1. The mean pedigree size and standard deviation as well as the total number of relative pairs were obtained in order to calculate the prevalence for AD, polyautoimmunity, and MAS as main traits. Analyses were restricted to FDR. When early-onset and late-onset families age and age of onset were compared, the difference was statistically significant (*p* value < 0.001) as expected given their autoimmune disorder characteristics.

In total 716 and 443 individuals were included for the analyses, for late-onset and early-onset families, respectively (Table 1). Late-onset families included 37% males and 63% females while early-onset presented 51% males and 49% females. Moreover, females represented the most affected ones in late-onset families while in early-onset the ratio of the affected was close to 1 : 1 (male : female). In early-onset families, there was only one individual presenting with MAS among the 102 affected individuals.

### 3.1. Familial Aggregation (*λ*_*R*_)

The distribution of relationship types and total number of study subjects included in this study is presented in Table 3. No two probands belonged to the same family. Pairs of relatives discordant or concordant for AD, polyautoimmunity, and MAS were calculated in order to examine the family aggregation. Overall, the data is composed of 876 parent-offspring pairs and 706 different sib-pairs broken down to sister-sister (*n* = 336), sister-brother (*n* = 64), and brother-brother (*n* = 306) pairs (Table 3).

The prevalence of AD, polyautoimmunity, and MAS for each pair of relatives (parent/offspring [P/O], sibling/sibling [S/S]) is disclosed in Table 3. Previously reported prevalence values for familial pairs for AD in healthy individuals were taken into account for the examination of aggregation (*K*_PO_ = 1.32%; *K*_S/S_ = 0.91%) [21]. Also, using a putative chosen prevalence for all AD taken together as trait (*K*_pop_ = 2.5%), *λ*_*R*_ were calculated (Table 3). Values supporting familial aggregation (*λ*_*R*_ > 1.0) were observed for AD in late-onset families in P/O (*λ*_HI_ = 4.76, *λ*_pop_ = 2.51) and S/S (*λ*_HI_ = 13.39, *λ*_pop_ = 4.87) pairs, with the highest familial aggregation within sister-pairs (*λ*_HI_ = 21.91, *λ*_pop_ = 7.98). For polyautoimmunity, familial aggregation was not observed for P/O pairs but for S/S pairs (*λ*_HI_ = 3.58, *λ*_pop_ = 1.30). In early-onset families, familial aggregation was observed for AD in P/O (*λ*_HI_ = 1.37) and in S/S (*λ*_HI_ = 4.04, *λ*_pop_ = 1.47). No aggregation for MAS was observed in any pair of relatives.

### 3.2. Segregation Analysis

The parameter estimates and test statistics from the segregation analyses for late- and early-onset families for AD, polyautoimmunity, and MAS are presented in Tables 4 and 5, respectively.

To determine support for familial or residual association in the data, initially we compared four no-transmission models, each having different type of familial association, to inspect whether the sibling (S) correlation equals the parent-offspring correlation (FO and/or MO, F: father, M: mother, and O: offspring). Four no major models were fitted and compared; each, respectively, assumed (1) *ρ*FO; *ρ*MO; *ρ*SS-free; (2) *ρ*FO = *ρ*MO, *ρ*SS-free; (3) *ρ*FO = *ρ*MO = *ρ*SS; and (4) *ρ*FO = *ρ*MO = *ρ*SS = 0 (the no multifactorial component model). *ρ*FM was assumed to be 0 for all models. The model where both parent-offspring and sibling residual associations are equal (i.e., *ρ*FO = *ρ*MO = *ρ*SS) fitted the data better than any of the other three models for AD, polyautoimmunity, and MAS for both late- and early-onset families (results not shown), thereby providing support for the existence of familial association in the data and inclusion and estimation of familial association parameters in the subsequent models. To determine whether sex should be included in the segregation models, two nontransmission models were initially fitted, one including the covariate and the other not, and then compared by AIC. Results showed that including sex as a covariate in the models allowed better model fitting (data not shown).

The hypothesis of no major gene was tested by comparing the random environmental (Model 1) and general transmission model (Model 9) (Table 2). The random transmission model was rejected in late-onset disease families, supporting the existence of a major gene in AD (*p* < 0.05, AIC = 708.08), polyautoimmunity (*p* < 0.05, AIC = 501.61), and MAS (*p* < 0.05, AIC = 296.46) (Table 4), while in early-onset families the model could not be rejected (*p* = 0.55, AIC = 438.29) (Table 5). Subsequently, the major gene hypothesis was further tested by comparing the major gene only model (Model 8) and the general transmission model (Model 9) (Table 2). For this comparison, the hypothesis for the major gene was rejected only for AD in late-onset families (*p* < 0.05, AIC = 679.08) (Table 4), while it was not rejected for late-onset families when taking polyautoimmunity and MAS as main traits, as well as in early-onset families for AD (Table 5). Of note, for early-onset families due to low frequency of polyautoimmunity and MAS, only models for AD as a main trait were estimated.

After having procured evidence for the segregation of major gene(s) in late-onset families with AD as the main trait and not for polyautoimmunity and MAS for late-onset and for AD in early-onset families, the hypothesis of Mendelian transmission was tested by comparing the Mendelian proposed models (Models 2, 4, 6, and 8) with the general transmission model (Model 9) (Table 2). Dominant, recessive, codominant, and additive Mendelian transmission models were rejected for late-onset families when taking AD as a trait. All the same, when a multifactorial/polygenic parameter was added to the dominant and recessive Mendelian models (Models 3 and 5, resp.) and compared with the Mendelian counterpart without the multifactorial component, no change in the rejection of the models was observed (Table 4).

## 4. Discussion

The commonality between ADs is the damage to tissues and organs arising from the loss of tolerance and in most cases a gender imbalance [46]. Research generally focuses on a single disease, although autoimmune phenotypes could represent pleiotropic outcomes of nonspecific disease genes underlying similar immunogenetic mechanisms [47]. While it is apparent that multiple cases of a single disease cluster within families [4], more striking are the individuals in those families afflicted with multiple ADs [3].

This report presents the familial aggregation and segregation analyses of AD, polyautoimmunity, and MAS in Colombian families. We have analyzed 210 families (i.e., 127 late-onset diseases and 83 early-onset ones) in Table 1, for which a total of 716 and 443 individuals were analyzed (Table 1). Each pedigree was ascertained through an affected proband fulfilling the inclusion criteria presented in Section 2. This study is restricted and takes into account AD, polyautoimmunity, and MAS as main traits presented in the recruited families (Figure 1). The recruited families were divided into two types of family given by the pathology presented in the proband (i.e., early-onset families are constituted mainly by T1D probands and late-onset families by AD known to develop later in life). Results show differences between late- and early-onset families regarding their age, age of onset, and sex distribution, which is expected given the particular and specific autoimmune disorder prevalence (Table 1, Figure 1).

Analyses of familial aggregation treat the family like any other unit of clustering. In addressing whether there is phenotypic aggregation within families, no attempt is made to determine the cause of any aggregation [48]. The observation and portrayal of familial autoimmunity and the outline of MAS have put aside the environmental aggregation and given a greater value towards the common/rare genetic component for diverse autoimmune phenotypes with a generally common background [4]. When considering the familial aggregation of AD, polyautoimmunity, and MAS for both types of families, values supporting the aggregation of AD in late- and early-onset families for P/O and S/S pairs, with the highest aggregation observed between sister-pairs of late-onset families, were observed (Table 3). For polyautoimmunity as a trait only aggregation was observed between S/S pairs in late-onset families. No familial aggregation for MAS was observed for any type of family. This suggests and confirms that polyautoimmunity and MAS are not AD independent traits and that gender, age, and age of onset represent factors that define and allow the study of the dynamics of the traits within the familial group.

Segregation analyses help to assess the possible genetic mode of segregation of a trait by consideration of relevant hypothesis-based mathematical models. Findings from segregation analyses are often used to formulate tailored research hypotheses for the trait under investigation and/or to decide the type of investigative effort to be put forward. This study was carried out to assess types of familial dependence in AD, polyautoimmunity, and MAS to investigate possible evidence of transmission of major gene(s) and to determine the best mode of transmission for such major gene(s). The presented analyses indicate evidence for the familial transmission of major gene(s) with no clear discernible classical known Mendelian transmission in late-onset families when AD is taken as the main trait, while for polyautoimmunity and MAS familial transmission fails to be demonstrated. In early-onset families analyses did not demonstrate a major gene effect but a random environmental model explaining the presence of the phenotypes in the families. These results thus provide evidence for the genetic role in the etiology of AD in late-onset families by showing support for major gene(s) mode of segregation of susceptibility to AD, while for the early-onset families and perhaps by their relatively young status eludes a clear picture of autoimmunity segregation and aggregation in these families.

Previous segregation analyses have proposed models in families with more than one member affected by autoimmune hemolytic anemia and chronic thrombocytopenic purpura compatible with a Mendelian dominant trait [49]. In African Americans [50, 51] and EA [52] SLE families, presenting FAD, a dominant inheritance is reported, while in Chinese families segregation analyses describe a polygenetic model and major gene model, suggesting a polygenetic multifactorial disease [53]. Other analyses in VIT for Chinese families suggest a dominant inheritance model [54], while other reports suggest a non-Mendelian pattern supporting a multifactorial, polygenic inheritance [38]; even so other models describe a major dominant gene and the existence of strong environmental effects acting on a recessive genotype [25]. More generally, a Mendelian dominant genetic inheritance is proposed in many ADs, like SS [55] and T1D [56], while segregation is better explained by either dominant or codominant or polygenic models in APS [57], RA [26], and idiopathic inflammatory myopathies [58]. Others suggest that several major ADs result from pleiotropic effects of a single major gene on a polygenic background [26]. Finally, in traits such as MS segregation results are indeterminate and cannot be explained by a genetic model [59].

## 5. Conclusions

Overall, aggregation and segregation analyses in Colombian families enriched by autoimmunity as a trait show how ADs, polyautoimmunity, and MAS are not independent entities. Familial aggregation for ADs was observed between parents and offspring as well as in sibling pairs in late-onset families, while aggregation for polyautoimmunity and MAS was lesser given by the fact that both traits represent a more complex etiology with lower prevalence but still a common autoimmunity background. Segregation analyses were not able to discern a Mendelian transmission model but still suggested major gene(s) transmission for AD in late-onset families, while for early-onset families a stochastic model was suggested. Thus, a clinical defined individual AD, defined by symptoms and signs, might not be completely juxtaposed to the AD trait defined by environment and genetics, which makes the task to define and untangle disease mechanisms even more difficult. Last but not least, to further study and describe the familial dynamics of two or more cluster ADs, approaches such as familial coaggregation might find their place towards the exploration of common familial factors on top of studies taking into account AD, polyautoimmunity, and MAS as a trait in order to disentangle the common/rare genetic landscape of autoimmunity.

## Figures and Tables

**Table 1:** Characteristics of probands and families classified as late-onset and early-onset.

Characteristic	Late-onset				Early-onset		
	All	AD	PolyAD	MAS	All	AD	PolyAD/MAS
Age (yrs)	49.43	45.99	45.49	44.81	32.32^*∗∗*^	19.54^*∗∗*^	16
[Min, Max]	[11, 91]	[13, 83]	[16, 78]	[20, 64]	[3, 94]	[4, 70]	
Age at onset (yrs)	—	32.80	33.42	33.97	—	7.77^*∗∗*^	11
[Min, Max]		[5, 62]	[5, 62]	[5, 62]		[1, 24]	
Male							
Aff (Unaff)	265	24 (156)	8 (172)	2 (178)	227	50 (141)	0 (191)
Female							
Aff (Unaff)	451	195 (216)	91 (320)	41 (370)	216	52 (152)	1 (203)
Number of Peds	127				83		
Mean size ± SD	5.64 ± 2.76				5.34 ± 2.94		
[Min, Max]	[3, 16]				[3, 20]		

AD: autoimmune disease; PolyAD: polyautoimmunity; MAS: multiple autoimmune syndrome. Data correspond to FDRs affected or unaffected and taking into account the analysis. Aff: affected; Unaff: unaffected.

^*∗∗*^ *p* value < 0.001 *t*-test when comparing late-onset versus early-onset variables.

**Table 2:** Parameter estimates from segregation analysis of autoimmune disease proband-ascertained pedigrees.

Model/parameter	* *Type * *susceptibilities			Transmission probabilities			Freq	Multifactorial/polygenic effect^b^
	*β*_AA_	*β*_AB_	*β*_BB_	*τ*_AA_	*τ*_AB_	*τ*_BB_	*q*_A_	*ρ*_FM_ = 0^a^; *ρ*_F0_ = *ρ*_M0_
(1) Random environmental	—	—	—	*q*_A_	*q*_A_	*q*_A_	*∗*	0
(2) Dominant	*∗*	*β*_AA_	*∗*	1	0.5	0	*∗*	0
(3) Dominant multifactorial	*∗*	*β*_AA_	*∗*	1	0.5	0	*∗*	*∗*
(4) Recessive	*∗*	*β*_BB_	*∗*	1	0.5	0	*∗*	0
(5) Recessive multifactorial	*∗*	*β*_BB_	*∗*	1	0.5	0	*∗*	*∗*
(6) Codominant	*∗*	*∗*	*∗*	1	0.5	0	*∗*	0
(7) Additive	*∗*	(1/2)(*β*_AA_ + *β*_BB_)	*∗*	1	0.5	0	*∗*	0
(8) Mayor gene	*∗*	*∗*	*∗*	*∗*	*∗*	*∗*	*∗*	0
(9) General transmission^c^	*∗*	*∗*	*∗*	*∗*	*∗*	*∗*	*∗*	*∗*

^*∗*^Parameters freely estimated within an appropriate range; *q*_A_: allele frequency; when *τ*_AA_ = 1.0, *τ*_AB_ = 0.5, and *τ*_BB_ = 0.0, Mendelian transmission is assumed; when *q*_A_ is estimated under Mendelian transmission, Hardy-Weinberg proportions (*ψ*_AA_ = *q*_A_^2^; *ψ*_AB_ = 2*q*_A_^2^(1 − 2*q*_A_^2^); *ψ*_BB_ = *q*_B_^2^) are assumed.

^a^Father-mother correlations, set to 0 assuming absence of assortative mating or consanguineous mating.

^b^Polygenic transmission effect inclusion assumes that the phenotype is determined by polygenic inheritance, so the phenotype has one distribution, and familial correlations can explain the familial aggregation of the trait.

^c^All parameters are estimated in Model 9. As a result, all other models are nested, and thus the general model is used as the baseline to compare all other models in this study.

*Models Description.* Random environmental model (Model 1) assumes that the trait segregation is caused purely by a random environmental factor and there is no transmission from generation to generation (*τ*_AA_ = *τ*_AB_ = *τ*_BB_ = *q*_A_). Pure major locus transmission models (Models 2, 4, 6, and 8) assume major locus transmission in a Mendelian mode, without multifactorial/polygenic inheritance. Major gene plus multifactorial/polygenic models (Models 3 and 5) assumes that both a major locus (transmitted in a Mendelian mode) and a multifactorial/polygenic effect influence the trait. The general model (Model 9) is the unrestricted full model, which subsumes all of the other models.

**Table 3:** Familial aggregation (*λ*_*R*_) of autoimmune disease (AD), polyautoimmunity, and multiple autoimmune syndrome (MAS) in late-onset and early-onset families.

Type of family	Pairs of relatives	Total pairs	Pairs	*K* (%)	*λ*_*R*_ = *K*_*R*_/*K*_HI_	*λ*_*R*_ = *K*_*R*_/*K*_pop_
Late-onset	AD			*K*_AD_	*λ*_HI_	*λ*_pop_

	Parent/offspring	876	55/190/208	6.28	4.76	2.51
	Sibling/sibling	706	86/267/353	12.1	13.39	4.87
	Sister/sister	336	67/92/177	19.9	21.91	7.98
	Brother/brother	64	0/44/20	0.00	0.00	0.00
	Brother/sister	306	19/131/156	6.21	6.82	2.48

Late-onset	Polyautoimmunity			*K*_PolyAD_	*λ*_HI_	*λ*_pop_

	Parent/offspring	876	8/333/112	0.91	0.69	0.37
	Sibling/sibling	706	23/450/233	3.26	3.58	1.30
	Sister/sister	336	20/181/135	5.95	6.54	2.38
	Brother/brother	64	0/59/5	0.00	0.00	0.00
	Brother/sister	306	3/210/93	0.98	1.08	0.39

Late-onset	MAS			*K*_MAS_	*λ*_HI_	*λ*_pop_

	Parent/offspring	876	1/403/49	0.11	0.09	0.05
	Sibling/sibling	706	4/581/121	0.57	0.62	0.23
	Sister/sister	336	3/260/73	0.89	0.98	0.36
	Brother/brother	64	0/60/4	0.00	0.00	0.00
	Brother/sister	306	1/261/44	0.33	0.36	0.13

Early-onset	AD			*K*_AD_	*λ*_HI_	*λ*_pop_

	Parent/offspring	498	9/199/155	1.81	1.37	0.72
	Sibling/sibling	245	9/130/106	3.67	4.04	1.47
	Sister/sister	61	3/30/28	4.92	5.40	1.97
	Brother/brother	60	2/33/25	3.33	3.66	1.33
	Brother/sister	120	4/67/53	3.33	3.66	1.33

Early-onset	Polyautoimmunity/MAS			*K*_MAS_	*λ*_HI_	*λ*_pop_

	Parent/offspring	498	0/361/2	0.00	0.00	0.00
	Sibling/sibling	245	0/244/1	0.00	0.00	0.00
	Sister/sister	61	0/61/0	0.00	0.00	0.00
	Brother/brother	60	0/60/0	0.00	0.00	0.00
	Brother/sister	120	0/123/1	0.00	0.00	0.00

^a^Affected/unaffected/discordant pairs.

^*∗*^ *K*_AD_, *K*_PolyAD_, and *K*_MAS_ = prevalence for AD, polyautoimmunity, and MAS, respectively. *K*_HI_ = prevalence for AD in healthy individual's pedigrees as previously reported (*K*_PO_ = 1.32%; *K*_S/S_ = 0.91%) [21]. *K*_pop_ = chosen prevalence for the general population. Recurrent risk ratio (*λ*_*R*_ = *K*_*R*_/(*K*_HI_ or *K*_pop_)), where *R* is the specific relative pair used (P/O = parent/offspring; SIB = sibling/sibling). The chosen population prevalence (*K*) for AD was considered as 25/1000 individuals [21]. Prevalence is given in percentages.

**Table 4:** Parameter estimates from segregation analyses of early-onset families. For details in each model check Table 2. AD: autoimmune disease; PolyAD: polyautoimmunity; MAS: multiple autoimmune syndrome; ND: model not able to maximize.

Model/parameter	*β*_AA_	*β*_AB_	*β*_BB_	*q*_A_	*ρ*_SS_	*τ*_AA_	*τ*_AA_	*τ*_AA_	Sex	−2ln⁡(*L*)	d.f.	*p* value	AIC
AD													
Random environmental	—	—	—	0.19	1.43	*q*_A_	*q*_A_	*q*_A_	2.36	698.079	3	<0.05	708.079
Dominant	1.21	*β*_AA_	−109	0.05	0.00	1.00	0.50	0.00	2.16	707.994	5	<0.05	**715.994**
Dominant multifactorial	1.00	*β*_AA_	−1.16	0.07	−0.06	1.00	0.50	0.00	2.19	706.492	4	<0.05	716.492
Recessive	−1.09	*β*_BB_	1.21	0.95	0.00	1.00	0.50	0.00	2.16	707.994	5	<0.05	**715.994**
Recessive multifactorial	−1.15	*β*_BB_	1.33	0.94	−0.05	1.00	0.50	0.00	2.24	706.653	4	<0.05	716.653
Codominant	−33.00	1.41	−1.21	0.06	0.00	1.00	0.50	0.00	2.35	707.529	5	<0.05	717.529
Additive	1.94	0.38	−1.18	0.10	−0.09	1.00	0.50	0.00	2.08	706.956	5	<0.05	716.956
Mayor locus	−0.73	1.54	−2.07	0.01	0.00	0.56	0.00	1.00	2.14	667.079	1	0.52	**679.079**
General transmission	−0.75	1.49	−2.07	0.02	−0.05	0.56	0.00	1.00	2.13	666.871		Ref.	680.871
PolyAD													
Random environmental	—	—	—	0.35	2.27	*q*_A_	*q*_A_	*q*_A_	2.30	491.607	3	<0.05	501.607
Dominant	1.50	*β*_AA_	−2.09	0.01	0	1	0.5	0	2.10	499.629	5	<0.05	**507.629**
Dominant multifactorial	−0.89	*β*_AA_	−2.21	0.08	−0.22	1	0.5	0	1.89	499.127	4	<0.05	509.127
Recessive	−2.09	*β*_BB_	1.51	0.99	0	1	0.5	0	2.10	499.629	5	<0.05	**507.629**
Recessive multifactorial	−2.29	*β*_BB_	−0.98	0.90	−0.24	1	0.5	0	1.90	499.135	4	<0.05	509.135
Codominant	−48.15	1.44	−2.09	0.01	0	1	0.5	0	2.11	499.614	5	<0.05	509.614
Additive	−0.65	−1.56	−2.47	0.24	−0.27	1	0.5	0	1.85	499.233	5	<0.05	509.233
Mayor gene	−2.02	−0.44	−17.60	0.04	0	0.86	0.00	1.00	2.09	472.191	1	<0.05	**484.191**
General transmission	−66.10	−1.05	−3.47	0.00	1.86	1.00	0.00	0.39	1.86	459.356		Ref.	471.356
MAS													
Random environmental	—	—	—	0.51	2.65	*q*_A_	*q*_A_	*q*_A_	2.73	286.846	3	<0.05	**296.846**
Dominant	ND	*β*_AA_	ND	ND	0	1.00	0.50	0.00	ND	ND	5		
Dominant multifactorial	−2.27	*β*_AA_	−4.32	0.25	−0.06	1.00	0.50	0.00	2.25	286.875	4	<0.05	296.875
Recessive	ND	*β*_BB_	ND	ND	0	1.00	0.50	0.00	ND	ND	5		
Recessive multifactorial	−4.84	*β*_BB_	−2.28	0.72	−0.05	1.00	0.50	0.00	0.72	286.856	4	<0.05	296.856
Codominant	−2.27	−2.27	−4.66	0.27	0	1.00	0.50	0.00	−0.98	286.838	5	<0.05	298.838
Additive	−2.27	−4.14	−6.07	0.66	−0.97	1.00	0.50	0.00	2.25	287.122	5	<0.05	297.122
Mayor gene	24.70	−14.29	−18.36	0.00	0	0.00	0.00	0.15	37.20	271.525	1	<0.05	**281.525**
General transmission	−152.53	−3.36	−1.91	0.96	4.42	0.42	0.12	0.99	1.95	260.304		Ref.	276.304

**Table 5:** Parameter estimates from segregation analyses of early-onset families. AD: autoimmune disease. For details in each model check Table 2.

Model/parameter	*β*_AA_	*β*_AB_	*β*_BB_	*q*_A_	*ρ*_SS_	*τ*_AA_	*τ*_AA_	*τ*_AA_	Sex	−2ln⁡(*L*)	d.f.	*p* value	AIC
AD													
Random environmental	—	—	—	0.01	−0.83	*q*_A_	*q*_A_	*q*_A_	−0.02	426.292	3	0.55	438.292
Dominant	−1.05	*β*_AA_	−1.05	0.02	0	1	0.5	0	−0.03	451.220	5	<0.05	459.22
Dominant multifactorial	−1.99	*β*_AA_	−1.05	0.08	0.01	1	0.5	0	0.01	441.228	4	<0.05	451.228
Recessive	−1.07	*β*_BB_	−1.05	0.00	0	1	0.5	0	−0.03	451.220	5	<0.05	459.22
Recessive multifactorial	−2.80	*β*_BB_	−1.04	0.32	−0.53	1	0.5	0	0.01	440.46	4	<0.05	450.46
Codominant	−2.78	−1.05	−1.08	0.29	0	1	0.5	0	0.01	440.408	5	<0.05	452.408
Additive	−1.17	−1.17	−1.17	0.10	−0.48	1	0.5	0	0.01	441.265	5	<0.05	451.265
Mayor gene	115.3	21.2	−2.68	0.00	0	0.3	0.0	0.1	0.54	400.587	1	<0.05	412.587
General transmission	−9.57	−0.71	−0.91	0.32	−0.84	0.20	0.33	0.34	−0.005	427.342	0		443.342
